# An exploratory study to investigate the association between age, physical activity, femoral trochlear cartilage thickness and biomarkers of tissue metabolism in adult males

**DOI:** 10.1007/s00421-021-04655-y

**Published:** 2021-03-13

**Authors:** Harry M. Roberts, Claire L. Griffith-McGeever, Julian A. Owen, Lewis Angell, Jonathan P. Moore, Jeanette M. Thom

**Affiliations:** 1 School of Sport, Health and Exercise Sciences, Physical Activity for Health and Well Being (PAWB) Research Group, Bangor University, Bangor, UK; 2 School of Biosciences and Medicine, University of Surrey, The Leggett Building, Daphne Jackson Road, Guildford, GU2 7WG UK; 3 School of Medical Sciences, University of New South Wales, Sydney, Australia

**Keywords:** Ultrasound, Knee cartilage thickness, Age, Cartilage metabolism, Joint lubrication

## Abstract

**Purpose:**

To investigate the association between age, physical activity, femoral trochlear cartilage thickness and biomarkers of tissue metabolism in a cross-sectional sample of adult males. This study utilizes several emerging biomarkers that have been associated with early joint degenerative changes; serum COMP (cartilage oligomeric matrix protein), HA (hyaluronan) and lubricin.

**Methods:**

Eighty-one males (age: mean (range): 43(18–70) years; body mass index: 25.2 (21.0–30.6) kg/m^2^) volunteered. Resting serum COMP, HA and lubricin concentrations were determined via commercially available enzyme-linked immunosorbent assay (ELISA) and femoral trochlear cartilage thickness via supra-patellar ultrasound imaging. Physical activity levels were assessed using questionnaires. Statistical analyses were performed using correlation and regression analyses.

**Results:**

Age was correlated with lateral trochlear cartilage thickness (*r* = − 0.372; *p* < 0.01) and serum COMP (*r* = 0.342; *p* < 0.01). 7-day physical activity was correlated with serum COMP (*r* = 0.357, *p* < 0.01), and 12-month physical activity with both lateral trochlear cartilage thickness (*r* = 0.340, *p* = 0.01) and serum HA (*r* = 0.296, *p* < 0.05). Regression analyses revealed that age significantly accounted for the variability in lateral cartilage thickness and serum COMP, following the adjustment for potential cofounders. However, the association between age and lateral trochlear cartilage thickness was not moderated by physical activity levels (all *p* > 0.05).

**Conclusion:**

This study indicates that older age may be associated with thinner lateral trochlear cartilage and higher cartilage turnover. Being physically active may also be positive for lateral trochlear cartilage thickness. However, overall, both age and physical activity level only account for a small amount of the variability in cartilage thickness and serum biomarkers.

## Introduction

Understanding how age and physical activity are associated with joint cartilage may be crucial for the development and progression of cartilage atrophy and osteoarthritis (OA). Articular cartilage undergoes changes with age, which can increase the vulnerability to degenerative change (Martin and Buckwalter 2002). Studies have previously indicated that age is associated with surface fibrillation of tibial cartilage (Young et al. 2006), an increased number of defects (Ding et al. 2005), and a reduction in tibial cartilage volume and patellar and femoral cartilage thickness, including femoral trochlear cartilage (Hudelmaier et al. 2001; Hanna et al. 2005; Özçakar et al. 2014). In contrast, other studies exploring the relationship between physical activity and OA are inconsistent; cross-sectional studies have reported higher rates of knee OA in athletes (Kujala et al. 1995; Spector et al. 1996), some have shown that physical activity may have no effect (Felson et al. 2007), while others have found that physical activity may protect the knee joint from degenerative changes (Lane et al. 1993; Rogers et al. 2002). However, regular physical activity has been associated with increased tibial cartilage volume, a reduced number of tibiofemoral cartilage defects (Racunica et al. 2007), as well as a reduction in the rate of total volume cartilage loss across the knee joint (Foley et al. 2007).

While there is often some concern as to whether too much physical activity will lead to OA, engaging in regular physical activity is important for general health, reducing obesity, and may prevent premature death (Warburton et al. 2006). Moreover, many activities are known to benefit the joint and surrounding joint structures (Beckwée et al. 2013). However, several individual factors, including, age, sex, BMI, muscle strength, previous injury, and joint alignment, may mediate the relationship between physical activity and risk of developing knee OA (Urquhart et al. 2008). Physical activity is well documented to help modify and manage several of these independent risk factors of OA (Beckwée et al. 2013) and is also useful for reducing the symptoms and progression of OA in individuals who have already been diagnosed with the condition (Nelson et al. 2014). However, whether physical activity can moderate associations between age and cartilage remains unclear.

Progressive thinning of knee joint cartilage is characteristic of OA and assessment of cartilage thickness, therefore, is useful for monitoring disease progression (Wang et al. 2012). Several imaging techniques enable relatively easy assessment. MRI measures both cartilage thickness and volume across several plates with high-degree precision and reliability for determining change (Wang et al. 2012). Ultrasonography is also a valid and reliable method to assess femoral cartilage thickness, despite some limitations related to the narrow acoustic window (Naredo et al. 2009; Roberts et al. 2018). Furthermore, ultrasound provides a more readily accessible, inexpensive, clinically orientated option to assess femoral trochlear cartilage thickness for both diagnostic and research purposes (Naredo et al. 2009).

Biomarkers of tissue metabolism, such as those found in the blood, may compliment traditional imaging techniques, thus providing early-warning indicators of compositional changes at the joint level. The use of serum biomarkers may be particularly important given that alterations in cartilage composition are likely to occur earlier and possibly prior to declines in cartilage thickness (Li et al. 2013). Several promising biomarkers exist, including, serum cartilage oligomeric matrix protein (COMP), HA (hyaluronan), and lubricin. These markers are understood to reflect cartilage turnover (Saxne et al. 1992; Sharif et al. 1995), joint inflammation/synovial proliferation (Seebeck and Haima 2013), and joint lubrication, respectively (Roberts et al. 2019). For example, baseline levels of serum COMP have been found to be elevated following knee injury (Catterall et al. 2010), OA (Neidhart et al. 2000) and rheumatoid arthritis (Law et al. 2015); such increases may reflect a shift toward increased cartilage degradation. Furthermore, elevated serum HA has previously been associated with OA (Elliott et al. 2005). Moreover, several in vivo animal studies provide evidence to suggest that lubricin synthesis is downregulated in degenerative joints (Abusara et al. 2013), following anterior cruciate ligament injury (Elsaid et al. 2012) and in a meniscectomy-induced OA model (Young et al. 2006). Although a small number of studies have demonstrated some association between age and both serum COMP (Clark et al. 1999; El-Arman et al. 2010) and serum HA (Inoue et al. 2011), these associations are not well defined.

Further research is required to determine whether age and physical activity level are associated with morphological properties of cartilage and/or with certain biomarkers of tissue metabolism known to be related to degenerative change. Therefore, the aims of this study were to determine whether: (1) age is associated with serum COMP, HA lubricin, and/or femoral trochlear cartilage thickness in healthy individuals, and (2) physical activity level is associated with serum COMP, HA lubricin, and/or femoral trochlear cartilage thickness in healthy individuals, and/or (3) whether physical activity level moderates the association with age.

## Methods

The local institutional ethics committee (Bangor University, School of Sport, Health and Exercise Sciences, Academic Research Ethics Committee) approved the protocol for this cross-sectional study, which conformed to the latest revision of the Helsinki declaration, except for registration in a database. Written informed consent was obtained from all participants and a heterogeneous sample of eighty-one healthy men was recruited. Participants were required to be aged between 18 and 75 years with a BMI of < 30 kg/m^2^. Individuals who had sustained a knee injury or suffered from chronic knee pain within the last 5 years were not recruited. Participants were required to report a previous knee injury (> 5 years) if they had visited a medical professional and had needed to stop physical activity for a period of 2 weeks or more. However, due to the retrospective nature of this questionnaire, the specific details of the injury were often unknown or not reported.

Participants visited the laboratory on two separate occasions, with a minimum of 48 h between visits. For each visit, they were instructed to avoid exercise in the preceding 24 h. At visit one, participants completed a questionnaire relating to health and medical conditions, lifestyle, previous knee injuries and knee pain. During this initial visit, a resting venous blood sample was also obtained prior to the measurement of femoral trochlear cartilage thickness using ultrasonography. At the second visit, participants provided a second resting blood sample.

The current level of physical activity was assessed using the International Physical Activity Questionnaire (IPAQ) last 7-day long version (Craig et al. 2003). This questionnaire quantified the time spent being physically active in the last 7 days within the following domains: (a) leisure time physical activity; (b) domestic and gardening activities; (c) work-related physical activity; (d) transport-related physical activity. The responses were converted into a continuous measure of physical activity [metabolic equivalent (MET) min/week] as documented by Craig et al. (2003). Participants were also grouped categorically into either ‘low’, ‘moderate’ or ‘high’ levels of physical activity using public health guidelines (Pate 1995). Physical activity over the last 12 months was assessed using a modified version of the Measurement of a Person’s Habitual Physical Activity questionnaire (Baecke et al. 1982). This also evaluated physical activity across several domains, these included: (a) work-related physical activity; (b) sport-related physical activity; (c) leisure-related physical activity. These data were used to calculate a continuous measure in the form of a total physical activity index and participants were also grouped categorically into either ‘low’, ‘moderate’ or ‘high’ levels of physical activity.

Resting blood samples were collected via venepuncture of the antecubital vein following 30 of minutes seated rest. All blood samples were allowed to clot and subsequently centrifuged for 15 min (4 °C, 1000 *g*). Serum was immediately frozen to − 80 °C and stored until analysis. Samples from visit 1 and 2 were obtained at the same time of day to avoid the potential influence of circadian rhythms and were averaged to establish a robust baseline value. Commercially available sandwich ELISAs were used to measure serum COMP (Human COMP ELISA kit KA0021, Abnova Corporation, Taiwan), HA (Hyaluronic Acid ELISA Kit ABIN1873289, Cloud-Clone Corp, USA) and Lubricin [Human Proteoglycan4 (PRG4) ELISA kit CSB-E14124h, Cusabio Biotech Co, China). All assays followed the manufacturer's specifications and were performed within the institution and all samples were analysed in either duplicates with the average computed and used for further analysis. Mean intra-assay coefficient of variation was 5.4%, 8.7%, and 9.0% for serum COMP, HA, and Lubricin, respectively. Mean inter-assay coefficient of variation was 9.5%, 9.6%, and 11.4% for serum COMP, HA, and Lubricin, respectively. *R*^2^ curve fit was > 0.98 across all analyses. Between-sample variation (i.e. visit 1 vs visit 2) was acceptable for serum COMP (coefficient of variation = 10.3%), but considerable for serum Lubricin (19.4%) and serum HA (28.3%).

The ultrasound assessment of femoral trochlear cartilage thickness was performed using a 12 MHz linear-array probe (Esaote S.P.A. MyLab50 ultrasound, Firenze, Italy) following a period of 15–30 min of seated rest. The transducer was placed in a supra-patella transverse position, perpendicular to the bone surface and orientated to optimise the US image as previously described (Naredo et al. 2009; Özçakar et al. 2014; Roberts et al. 2018). All ultrasonography scans were performed by the same researcher. Cartilage thickness was determined as the distance from the thin hyperechoic line formed at the synovial space–cartilage border to the line formed at the cartilage–bone border and was used to measure cartilage thickness at the lateral facet, medial facet and trochlear notch (Özçakar et al. 2014; Roberts et al. 2018). All images were analysed using ‘ImageJ’ software (ImageJ, National Institute of Health, Bethesda, MD, USA). Prior to analysis, all images were de-identified by second researcher for blinded analysis and cartilage thickness of each image was measured in triplicate by a single blinded researcher. Previous research has demonstrated high test–re-test reproducibility (intraclass correlation coefficient, 0.779–0.843) in femoral cartilage thickness measurement (Roberts et al. 2018). Right femoral cartilage thickness was used for all data analyses as we previously found that femoral cartilage thickness did not differ between the right and left intercondyle notch and medial condyle, and side-to-side difference observed at the lateral condyle between the left and right knee was small (1.78 vs 1.88 mm, *p* = 0.04) (Roberts 2017).

Statistical analyses were performed using SPSS (SPSS v.20.0, Chicago, IL, USA). Normality of data was explored by visual inspection of Q–Q plots and through analysis of residuals. Pearson correlations (parametric data) and Spearman’s rank correlations (non-parametric data) were performed initially to examine the relationships between all baseline continuous variables. Hierarchical regression analyses were used subsequently to examine the associations between age and each of the dependent variables (Serum COMP and lateral trochlear cartilage thickness) following adjustment for BMI, past injury and physical activity. Step one consisted of BMI, past injury, and physical activity (7-day IPAQ or PA activity over the last 12 months) and step two consisted of the addition of age. The independent variables that were added to the models were based on current theoretical considerations, i.e. previous associations with OA, as well as results from initial correlation analyses. Bootstrapped analyses were used if model assumptions were violated and robust regression was required. Finally, follow-up moderation analyses were used for further analyses of significant models, i.e. when age predicted the dependent variable. The purpose of this additional analysis was to determine the moderating effect of physical activity on age and the dependent variable. As previously described, 7-day IPAQ score and PA of the last 12 months were categorised into ‘low’, ‘moderate’ and ‘high’ groups. The SPSS PROCESS syntax (Hayes 2012) was used to complete this moderation analyses. An interaction was deemed present if a significant interaction was observed. If the assumption of normality was violated (e.g. serum COMP), the data were transformed using the natural log function. Statistical significance was set as *p* < 0.05. Effect sizes were calculated using Cohen’s f2 method, with f2 ≥ 0.02, f2 ≥ 0.15, and f2 ≥ 0.35 representing small, medium, and large effect sizes, respectively (Cohen, 1988).

Prior to the study sample, size calculations were performed using PASS software (NCSS LLC, Kaysville, Utah) using serum lubricin as the outcome variable and age as the independent variable. A sample size of 594 was required at 80% power to detect a change in a change in slope from 0.00 under the null hypothesis to 0.10 under the alternative hypothesis, when the two-sided significance level was set as 0.05. However, to detect a change of in slope from 0.00 to 0.30, a more feasible sample size of 61 was specified. Given that limited literature exists to provide a clear indication of the expected relationship between the serum biomarkers and age, this study was termed ‘exploratory’ and targeted a recruitment total of a minimum of 60 participants.

## Results

Eighty-one males (age: mean (range): 43 (18–70) years) were included in this cross-sectional study. Physical characteristics and descriptive statistics for the outcome measure are shown in Table 1. Participants were healthy with no documented health conditions or disease. Previous knee injuries (sustained ≥ 5 years prior to enrolment) were reported in 32% (*n* = 26) of participants and consisted of a combination of soft tissue injuries to muscles around the knee joint (*n* = 4), tendons (*n* = 1), ligaments (*n* = 1), menisci (*n* = 1), or were undiagnosed (*n* = 19).

**Table 1:** Physical characteristics of participants

Variable	18–30 (*n* = 21)		31–40 (*n* = 20)		41–50 (*n* = 12)		51–60 (*n* = 13)		61–70 (*n* = 15)		Total (*n* = 81)		
	Mean	SD	Mean	SD	Mean	SD	Mean	SD	Mean	SD	Mean (SD)		Range
Age (years)	25	3	35	3	47	3	55	3	67	3	43	16	18—70
BMI (kg/m^2^)	23.9	1.9	25.9	2.8	24.8	2.3	27.0	1.9	25.4	2.0	25.2	2.4	21.0–30.6
7-day IPAQ (MET min/week)	3189	2010	2329	1737	4396	3684	3794	4653	7282	5515	4604	4882	400–24,216
12-month physical activity index	8.9	1.6	7.8	1.6	9.7	0.7	7.8	2.1	7.6	1.6	8.2	1.7	5.00–12.64
Number of reported injuries	7		7		2		3		7		26		
COMP (ng/ml)	687.14	167.94	683.87	209.83	769.02	201.15	877.52	373.35	899.43	426.85	766.69	288.27	450.70–2300.74
Serum Lubricin (ng/ml)	111.94	53.05	110.79	49.01	94.49	16.68	118.10	44.27	122.89	34.35	112.75	43.44	24.34–201.80
Serum Hyaluronan (ng/ml)	23.94	21.92	18.04	16.03	14.47	10.34	27.62	22.88	18.01	17.95	20.57	18.65	3.05–73.32
Trochlear cartilage (mm)													
Intercondyle notch thickness	2.29	0.45	2.22	0.50	2.09	0.28	2.00	0.29	2.20	0.43	2.18	0.42	1.28–3.38
Medial thickness	2.04	0.32	2.00	0.34	2.01	0.38	1.93	0.39	1.90	0.46	1.98	0.37	1.27–2.93
Lateral thickness	2.11	0.25	1.92	0.26	1.90	0.17	1.85	0.44	1.80	0.30	1.93	0.3	1.41 – 2.73

Results are shown as mean and standard deviation

*SD *standard deviation, *BMI *body mass index, *IPAQ *International physical activity questionnaire

Initial correlation analyses demonstrated that age correlated with lateral trochlear cartilage thickness (*r* = − 0.372, *p* < 0.001) and serum COMP (*r* = 0.342, *p* < 0.01). In contrast, there was no significant correlation between age and other trochlear cartilage locations, serum HA or serum lubricin. With reference to physical activity, 7-day IPAQ correlated with serum COMP (*r* = 0.357, *p* < 0.01), and 12-month physical activity correlated with lateral trochlear cartilage (*r* = 0.340, *p* = 0.01) and serum HA (*r* = 0.296, *p* < 0.05). All results from the correlation analyses are reported in Table 2.

**Table 2:** Correlation analyses

Variable	Age	BMI	7-day IPAQ	12-month physical activity	COMP	HA	Lubricin	Intercondyle notch thickness	Medial thickness	Lateral thickness
Age	1	0.292^**^	0.294^*^	− 0.223	0.342^**^	− 0.071	− 0.082	− 0.151	− 0.149	− 0.372^**^
BMI		1	− 0.061	− 0.204	− 0.001	− 0.117	− 0.168	0.051	− 00.145	− 0.463**
7-day IPAQ			1	0.342^*^	0.357^**^	0.145	0.254	0.032	0.200	0.075
12-month physical activity index				1	0.108	0.296^*^	− 0.082	0.091	0.340^*^	0.242
COMP					1	0.074	0.310^*^	− 0.081	− 0.109	− 0.145
HA						1	− 0.006	0.132	0.071	0.133
Lubricin							1	− 0.245	− 0.222	− 00.15
Intercondyle notch thickness								1	0.470^**^	0.381^**^
Medial thickness									1	0.420^**^
Lateral thickness										

*BMI *body mass index, *IPAQ *International physical activity questionnaire

**Correlation is significant at the 0.01 level (2-tailed); *Correlation is significant at the 0.05 level (2-tailed)

**Fig. 1:**
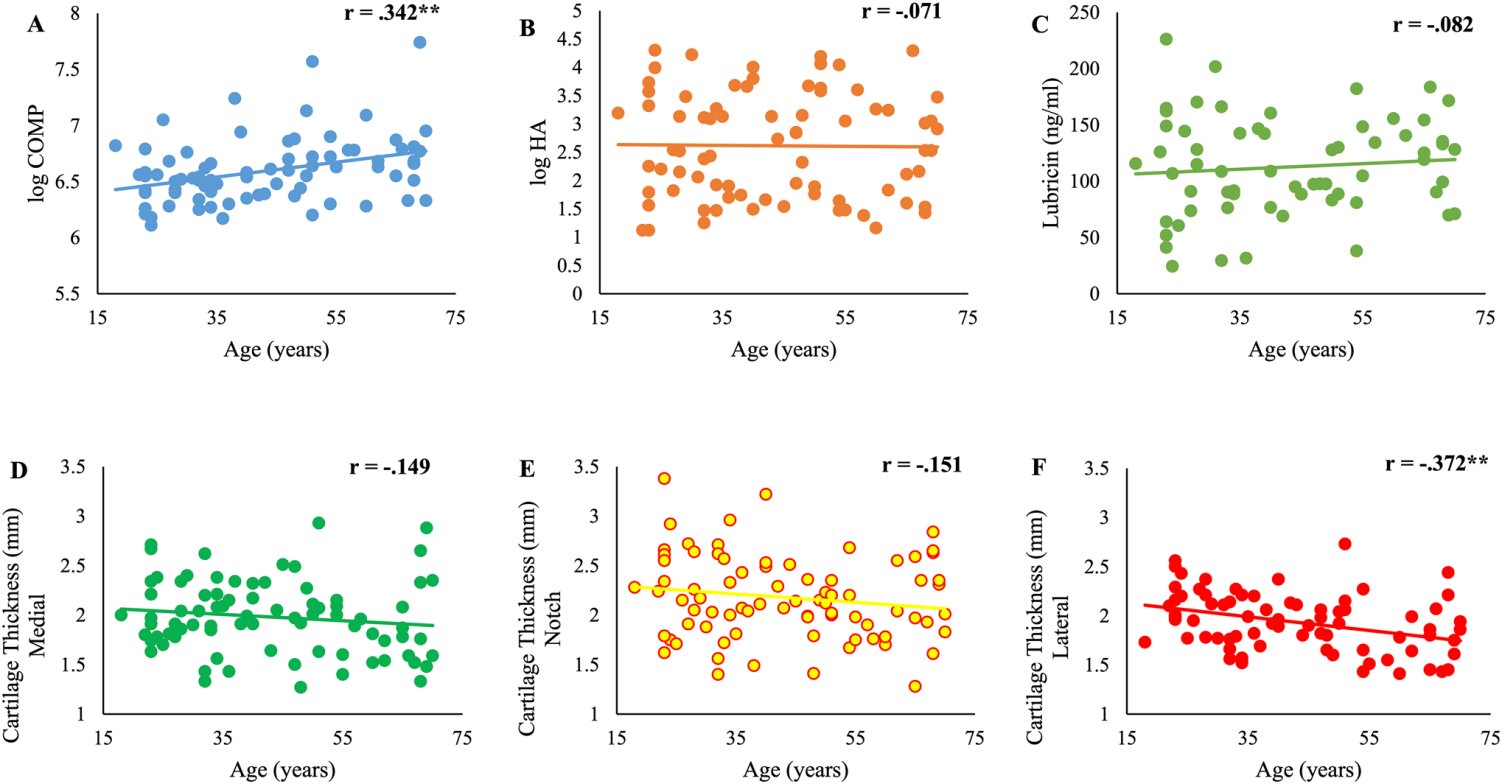
Correlation between age and dependent variables; **a** serum COMP; **b** serum HA; **c** serum lubricin; **d** Trochlear femoral cartilage thickness (medial); **e** Trochlear femoral cartilage thickness (notch); **f** Trochlear femoral cartilage thickness (lateral). **Significant at the 0.01 level; *Significant at the 0.05 level

Hierarchical regression analyses were used to further examine the relationship between age and lateral trochlear cartilage thickness. At step one, analysis revealed that 12-month physical activity, past injury and BMI significantly predicted lateral cartilage thickness (*R*^2^ = 0.259, *p* < 0.01). The addition of age at step two revealed that age accounted for 5.7% of the variability in lateral cartilage thickness (*R*^2^ change = 0.057, *p* < 0.05), over and above, the variability accounted for by BMI, past injury, and 12-month physical activity level. However, the robust bootstrapped analysis revealed that age accounted for only 2.7% of the variability in lateral cartilage thickness and did not significantly add to the model (*R*^2^ change = 0.027, *p* = 0.201). Effect size calculations indicate that age, as an individual predictor, only has a small effect on lateral cartilage thickness (Fig. 1).

Additional hierarchical regression analyses were used to further examine the relationship between age and serum COMP. At step one, analysis revealed that 7-day IPAQ, past injury and BMI did not significant predict serum COMP (*r* = 0.140, *p* = 0.05). Moreover, the addition of age at step two, did not significantly contribute to the model (*R*^2^ change = 0.062, *p* = 0.06). However, bootstrapped analysis also revealed that age significantly predicted the serum COMP (*R*^2^ change = 0.081, *p* < 0.05), over and above, the variability of the BMI, past injury, and 7-day IPAQ. Effect size calculations indicate that age, as an individual predictor, only has a small effect on serum COMP. A summary of the main hierarchical regression analyses is presented in Table 3.

**Table 3:** A summary of hierarchical regression analysis for each dependent variable

	Lateral cartilage thickness	Serum COMP
Independent variable		
Step 1		
BMI	− 0.326*	0.034
Past injury	− 0.147	0.029
12-month physical activity/7-day IPAQ	0.281*	0.371*
R	0.509	0.374
R2	0.259	0.14*
Step 2		
BMI	− 0.286*	− 0.093
Past injury	− 0.136	0.017
12-month physical activity/7 day IPAQ	0.234	0.277*
Age	− 0.248*	0.272
*R*	0.562	0.449
*R*2	0.316	0.202
∆*R*2	0.057*	0.062
Effect size (Cohen's F2)		
Full model (Step 2)	0.46	0.25
Age as individual predictor	0.06	0.07

12-month physical activity was used as a predictor for the analysis of lateral trochlear cartilage thickness and 7-day IPAQ was used as individual predictor for analysis of serum COMP

*IPAQ *International physical activity questionnaire

*Significant at the 0.05 level (2-tailed)

**Significant at the 0.01 level (2-tailed)

### Moderation analyses

**Fig. 2:**
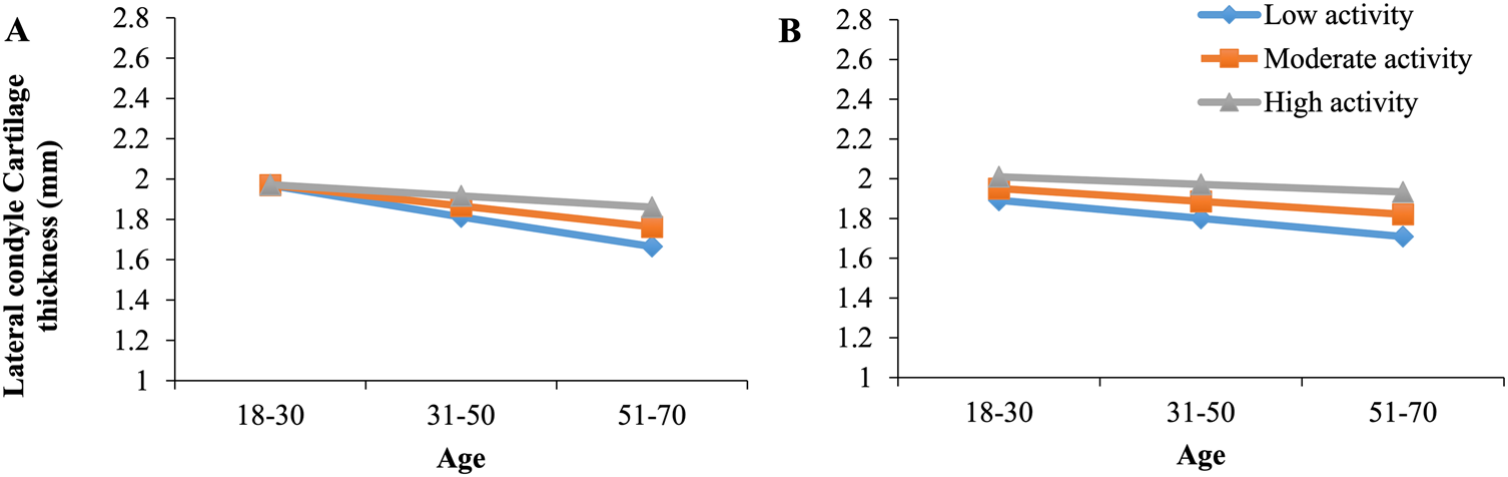
An interaction plot to demonstrate the moderating effects of physical activity on the association between age and lateral condyle cartilage thickness; moderating effects of **a** 7-day IPAQ score and **b** 12-month physical activity score

Result of the moderation analyses revealed that neither 7-day IPAQ [*b* = 0.0030, 95% (− 0.012, 0.0071), *t* = 1.44, *p* = 0.16] nor 12-month physical activity [*b* = 0.0012, 95% CI (− 0.0018, 0.0043), *t* = 0.79, *p* = 0.43], moderated the relationship between age and lateral cartilage thickness, i.e. there was no significant interaction effect. A graphical illustration of the interaction plot for the moderated regression analyses is provided (Fig. 2).

## Discussion

To our knowledge, this is the first study to evaluate associations of age and biomarkers of articular cartilage, and ultrasound measurements of femoral trochlear cartilage thicknesses in the same population. For a cohort of adult men studied here, age was associated with serum COMP and lateral trochlear cartilage thickness, which are consistent with knee articular cartilage atrophy with advancing age. Notably, physical activity levels for the previous 12 months associated positively with lateral femoral trochlear cartilage thickness, although higher levels of physical activity did not appear to moderate the association between age and lateral cartilage thickness.

Studies using ultrasound to investigate age and cartilage thickness are limited. However, age has previously been identified as a risk factor for decreased femoral trochlear thickness in men (Özçakar et al. 2014). Similarly, research using MRI also found associations between age and cartilage thickness at other sites across the knee joint, including medial tibial cartilage, lateral tibial cartilage and patellar cartilage (Ding et al. 2005). Cartilage thicknesses at multiple sites, including the femoral trochlear, have been found to be significantly thinner in asymptomatic elderly participants compared with asymptomatic young individuals (Hudelmaier et al. 2001). Such losses in cartilage thickness may be considered normal within the ageing process. In the current study, the association with age indicated that potential changes in cartilage thickness were small and limited to the lateral facet of trochlear cartilage. Although it is important to recognise the different techniques used and the different locations, the association observed by Ding and colleagues was similar to that observed in the current study (Ding et al. 2005). The current study also found differences in the age-related associations between the various locations of trochlear cartilage, with age associated with lateral trochlear cartilage thickness, but not within the notch or medial trochlear cartilage. Previous studies have also found that age was associated with changes at the lateral but not the medial trochlear facet (Özçakar et al. 2014). Differences in the thicknesses across the joint may relate to changes in contact pressure between the patellar and lateral facet. For example, small alterations in force and lateral patella malalignment are understood to impact the contact pressure on the lateral facet of the patellofemoral joint (Hinman and Crossley 2007). Moreover, differences across the patellofemoral joint are not uncommon as indicated by the fact that lateral compartment patellofemoral joint OA is more prevalent than medial compartment patellofemoral joint OA (Elahi et al. 2000).

Physical activity over the last 12 months and 7-day physical activity was positively associated with lateral trochlear cartilage thickness and serum COMP, respectively. These results suggest that physical activity may be important for regular joint metabolism and to protect against cartilage atrophy, and supports previous studies that have also demonstrated positive relationship between physical activity and cartilage morphology (Foley et al. 2007; Racunica et al. 2007; Özçakar et al. 2014). However, our results also indicate that physical activity does not moderate the age-associated decrease in cartilage thickness, i.e. low, moderate or high levels of physical activity did not alter the association between age and lateral cartilage thickness. Joint motion and load are understood to be important to maintain healthy cartilage (Fox et al. 2009). However, it was anticipated that potential benefits of physical activity may depend on the level of physical activity an individual completes. Previous research indicates that the benefits of physical activity for cartilage morphology may be most prominent following moderate levels of physical activity, which avoids high-impact or excessive knee bending (Lin et al. 2013; Virayavanich et al. 2013). There is also evidence that conditions of underloading/immobilisation may initiate catabolic processes in cartilage metabolism and be related to cartilage thinning (Vanwanseele et al. 2002; Liphardt et al. 2018). Previous work found that participation in vigorous physical activity, which was predominantly weight‐bearing in nature, was associated with a reduction in patella cartilage loss and a trend toward a reduced risk for worsening patella cartilage defects (Teichtahl et al. 2009); however, these benefits were not observed among those with already established cartilage defects. In a similar study, MRI T2 relaxation times were found to be no different between physical activity levels for individuals without OA risk factors. However, relaxation times were higher for individuals with OA risk factors, suggesting some cartilage degeneration (Hovis et al. 2011). Together, these results indicate that underlying joint health and/or additional risk factors may be crucial for benefits to be observed. Notably, around half of the cohort for the present study reported ‘high’ levels of physical activity (i.e. > 3000 MET min/week), and had limited risk factors, which may explain why physical activity did not have a stronger association with cartilage thickness.

Identifying associations with age is clinically important given that age is a key risk factor of OA. Changes in serum COMP may have prognostic significance and elevations in serum COMP among individuals with OA and have been associated with clinical features and symptoms (Clark et al., 1999). Further research is required to ascertain whether thinner cartilage or higher cartilage metabolism (of the magnitude observed in the current study) results in increased risk of future OA. Further work is also required to establish whether a serum COMP / trochlear cartilage thickness threshold can be identified, which may enable the identification of “cartilage thinning” or “elevated cartilage turnover” and individuals who may thus be at risk of OA or have early signs of OA. Such information could be extremely useful for the management of joint disease. Although not an aim of the study, this research has added value by providing additional normative data in this area. The serum biomarkers (COMP, HA and lubricin) and femoral cartilage thicknesses in the present study demonstrated considerable variability across individuals. As expected, the baseline serum COMP concentrations in the men studied here (Table 1) were lower when compared to those that have been observed previously in OA patients (767 vs 890–4100 ng/ml) (Jordan et al. 2003; Senolt et al. 2005). Similarly, baseline serum HA concentrations in the present study were also lower than previously reported in OA (21 vs 258 ng/ml) (Criscione et al. 2005). However, both serum COMP and HA concentrations were comparable to previously reported serum COMP values (767 vs 806 ng/ml) and serum HA (21 vs 41 ng/ml) in healthy individuals (Jordan et al. 2003; Wakitani et al. 2007).

While this current study provides new insight into several serum biomarkers and the relationship with age and physical activity, some methodological considerations warrant discussion. First, the use of ultrasound for the assessment of femoral cartilage thickness does have limitations. This technique is limited to the femoral trochlea, and while it does offer a high degree of reliability, it does not offer the accuracy of other imaging modalities and the results are not necessarily representative of the entire cartilage plate. These limitations are important to recognise given that associations observed with age in the present study are small and thus subtle changes may not be identified. Second, COMP, HA and lubricin all are systemic biomarkers expressed by several different tissues and not produced exclusively within the knee joint; thus, the serum values may also reflect synthesis from other tissues in the body. Third, the serum lubricin ELISA assay used detected not only the lubricin protein, but also several post-translational modifications of the PRG-4 gene. Moreover, it is also important to acknowledge the limitations in accuracy and reproducibility of both the serum lubricin and serum HA ELISA’s used in the current study. Finally, we attempted to strengthen our study and control for potential variations between sex and obese individuals, by limiting our sample to men who had a BMI to < 30 kg/m^2^, and to those who had not been recently injured. As a result, future studies should build on this initial exploratory research to investigate whether associations differ between women, those who are obese, and to further investigate the role of injury.

## Conclusion

The results of the current study demonstrate that age was associated with lateral trochlear cartilage and serum COMP. This indicates that older age may be associated with thinner lateral trochlear cartilage and higher cartilage turnover. In contrast, age was not associated with serum HA, lubricin or other cartilage locations. The positive association between physical activity and lateral trochlear cartilage also provides evidence to suggest that engaging in physical activity may be positive for lateral trochlear cartilage thickness. Notably, advancing age and physical activity account only for a small amount of the variability in cartilage thickness and serum biomarkers; therefore, other environmental factors and genetics may be responsible for the additional variation in this study. Nevertheless, this is new knowledge of how age and physical activity are associated with joint cartilage, which may be important in better understanding the development and progression of cartilage atrophy and OA.

## Abbreviations

BMI: Body mass index
COMP: Cartilage oligomeric matrix protein
ELISA: Enzyme-linked immunosorbent assay
HA: Hyaluronan
IPAQ: International physical activity questionnaires
MRI: Magnetic resonance imaging
MET: Metabolic equivalent
OA: Osteoarthritis
PRG4: Proteoglycan 4
